# Uncertainty and Fears Around Sustainability: A Qualitative Exploration of the Emotional Reactions of Dental Practitioners and Dental Care Professionals During COVID-19

**DOI:** 10.3389/froh.2021.799158

**Published:** 2022-01-21

**Authors:** Jennifer Knights, Laura Beaton, Linda Young, Mariana Araujo, Siyang Yuan, Jan Clarkson, Gerry Humphris, Ruth Freeman

**Affiliations:** ^1^NHS Education for Scotland, Edinburgh, United Kingdom; ^2^Dental Health Services Research Unit, School of Dentistry, University of Dundee, Dundee, United Kingdom; ^3^School of Medicine, University of St Andrews, St Andrews, United Kingdom

**Keywords:** COVID-19, sustainability, anxiety, uncertainty, dental training, primary care team

## Abstract

**Introduction:**

Recent cross-sectional surveys have shown the detrimental impact of COVID-19 on the health and well-being of dental practitioners and dental care professionals. This qualitative study complements the growing quantitative evidence base with an in-depth exploration of the lived experiences of those working in primary care dental teams in Scotland.

**Methods:**

Focus groups were carried out with primary care dental team members and trainees between July and October 2020. Olsen's tripartite framework of health service sustainability was operationalised to explore how participants experienced uncertainty and their attempts to sustain dental services.

**Results:**

Analysis revealed significant concerns surrounding the sustainability of dental services and dental training programmes as a consequence of the emergency level response to the pandemic. Restrictions on dentistry were seen to be severely impeding desirable clinical outcomes, particularly for the most vulnerable groups. Participants experienced being unable to deliver high quality care to their patients as both confusing and distressing. The capability of the dental health care system to meet a growing backlog of dental need and manage this effectively in a pandemic era was called in to serious question. Ongoing uncertainties were affecting how participants were thinking about their professional futures, with stress about income and employment, along with heightened experiences of professional isolation during the pandemic, resulting in some looking at possibilities for retraining or even considering leaving their profession altogether.

**Discussion:**

The impact of the pandemic has produced considerable uncertainty regarding the sustainability of dental services in the medium to longer term. It has also served to expose the uncertainties practitioners grapple with routinely as they attempt to sustain their NHS dental service delivery.

**Conclusion:**

This study brings in to sharp focus the diversity of challenges, confusions and uncertainties experienced by dental practitioners and dental care professionals during the COVID-19 pandemic and the need for suitable and ongoing measures to be put in place to support their mental well-being.

## Introduction

The COVID-19 pandemic has had a detrimental impact on the health and well-being of dental practitioners and dental care professionals. In particular, increased levels of psychological distress associated with fears of contracting COVID-19 from patients, anxiety about transmitting the virus to family members, and high levels of concern about the financial viability of practice and professional futures [1–3]. Collin et al. found psychological distress to be lower in dentists in the United Kingdom (UK) during the first national lockdown between late March and June 2020 when compared to previous research using the same measure. However, while some dentists valued remote working as an opportunity to re-evaluate their lives and careers, this was not the case for all. The highest levels of psychological distress were reported by general dental practitioners in England and those with a mixed NHS (National Health Service)-private practice commitment [4].

While most studies have focused upon dental practitioners, some recent research has examined the impact of the pandemic on the wider dental team. Mahendran et al. surveyed members of the dental team working within a large dental teaching hospital in the UK and found that the highest anxiety experienced was among dental nurses, who represented over 50% of those redeployed to other localities, followed by dentists in training grades who experienced disruption to their training [5].

Specifically examining health and well-being as an outcome of the uncertainties surrounding COVID-19 in Scotland, Humphris et al. surveyed dental practitioners and dental care professionals working in a primary care dental setting. Twenty-seven percent of the participating primary care dental staff reported depressive symptoms and 55% experienced emotional exhaustion. Moreover, compared with their trainee counterparts qualified primary care dental staff felt less prepared for managing their health, coping with uncertainty and financial insecurity [6].

Research during the 2003 SARS outbreak suggested that high-risk health care workers denied their psychological distress during the outbreak, but experienced detriment to their longer-term mental health functioning, including post-traumatic stress disorder [7]. Studies looking at the impact of the COVID-19 pandemic suggest that those in most need of psychological support, those at high exposure to the virus or with pre-existing mental health problems, are also those least likely to request or receive it [8]. Therefore, identifying who appears vulnerable and how they react to the pressures created by the COVID-19 pandemic in both the short and longer term, is critical for policymakers and educators in order that suitable and ongoing support can be designed and delivered.

Identifying and supporting individuals who are struggling is of course imperative, however, any attempt to understand how uncertainty and anxiety is experienced within the professions needs to be considered in terms of the attempts made to sustain dental services. Dentistry is an open complex system, sustained organizationally through the long-term ability to mobilize resources for dental care to meet public demand and need. We not only understand the notion of sustainability as the general continuation and maintenance of current service provision with associated desirable outcomes but also in terms of an ongoing process to adapt, develop and initiate change in response to the emerging needs of the system [9]. Olsen groups the determining factors of sustainability in to three clusters for analyzing health services: contextual factors, activity profile and organizational capacity. It is the interplay between these clusters and the capability to deliver at each level over time, and within a given context, that ensures that a health care system is sustainable [10]. We propose, therefore, that the concept of health service sustainability as operationalised by Olsen may be used to explore how dental practitioners and dental care professionals experience uncertainty and their attempts to sustain dental services.

The aim of this research was to explore qualitatively how the COVID-19 pandemic affected dental practitioners and dental care professionals in training and in primary care teams in terms of their lived experiences of uncertainty and attempts to sustain dental services. This qualitative exploration forms part of the research programme, CAREER [11], which is seeking to understand how the COVID-19 pandemic is affecting the anxieties, feelings of uncertainty and preparedness for practice of dental practitioners and dental care professionals working in primary care teams within Scotland.

## Methods

### Study Design

The design was a qualitative cross-sectional study using focus groups for data collection.

### Sample and Recruitment

All focus group participants had previously participated in a baseline survey as part of the CAREER programme (see Supplementary Material). Dental trainees were invited to participate in the survey via their training programmes in mid-June 2020; primary care dentists and dental care professionals were contacted in August 2020 via the NHS Educational for Scotland (NES) Portal, an online course booking system where dental professionals can opt in to receive marketing communications. The survey provided respondents with the option to volunteer to participate in a focus group which would involve discussing their anxieties and concerns regarding their current job role during the COVID-19 pandemic and their future career. The sample was therefore purposive, with final sample size determined by expression of interest in volunteering and subsequent availability to join a focus group from proposed dates and times.

### Data Collection

Focus groups were selected as the most appropriate tool for exploring the lived experiences and perspectives of our participants. Focus groups allow the researcher to tap into the cultural values, group norms and different types of knowledge produced through group interaction [12] and have been found to be more successful than individual interviews in encouraging personal and sensitive disclosures [13]. We were also interested in whether they would reveal divergences in opinion and the extent to which certain issues would recur across the groups [14]. To ensure a safe environment within which participants would feel comfortable to share their anxieties and concerns, the number of group participants was limited to between 5 and 8 in order that all would have sufficient opportunity to express their views, and the discussions were facilitated by RF, chief investigator of the CAREER programme and an experienced researcher and mental health specialist. Support was provided by members of the research team (JK, LB, MA, SY).

The focus groups were conducted and recorded online using Microsoft Teams. Semi-structured interview questions and prompts were used from a pre-agreed interview schedule informed by earlier group sessions held by two of the authors (RF and GH) with a volunteer sample of 12 trainee dentists from Scotland during February to April 2020, and through discussion with the CAREER project team. The areas of discussion included anxieties, worries and concerns regarding current job roles and future careers, maintaining quality care etc. during the COVID-19 pandemic.

The research team recognized the inevitable effect of researcher position and perspective and endeavored to maintain ongoing reflexivity throughout by critically engaging with one another's preconceptions, professional and personal thoughts and experiences in advance of each focus group and through holding a debrief immediately following each [15]. The inter-organizational and highly collaborative nature of the CAREER programme also allowed for the pooling of different areas of expertise and perspectives.

### Data Analysis

The focus group discussions were transcribed and transcripts pseudonymised and analyzed using a thematic analytical approach [16]. The transcripts were read and re-read by members of the research team (JK, LB, MA, and SY) and initial codes generated to systematically identify interesting and salient features of the data. These codes were collated and developed into potential themes within each focus group. Through group discussion, common and shared incidents, experiences and behaviors across the focus groups were distilled and categorized into themes and subthemes. This process acted to ensure trustworthiness in terms of credibility [15, 17].

We adopted Olsen's tripartite framework to explore how the identified themes and subthemes could be understood to operate in terms of contextual factors (e.g., COVID-19 and policy), activity profile (e.g., patient through-put, coping with patient and colleague demands, reduction in services offered) and organizational capacity (e.g., division of labor, workload, physical safety, values and culture, development and reward). This allowed for an in-depth exploration of how pandemic and policy factors have affected dental services and clinical practice, and how dental practitioners and dental care professionals made sense of the uncertainty experienced in their work and lives during the first 6 months of the pandemic. We acknowledge that the adoption of Olsen's clusters are artificial distinctions, nevertheless, we contend that incorporating this analytic approach provides a means of appreciating the external (e.g., policy change) and internal (e.g., coping with anxieties) issues associated with sustaining dental services during the current pandemic.

## Results

Six focus groups (three with trainees; three with primary care dental team members) took place between July and October 2020 (Table 1). Thirty-five participants took part, with between 5 and 8 participants per group. Forty-six percent (*n* = 16) were in postgraduate training [vocational dental practitioners (VDPs), vocational dental therapists (VDTs), core trainees (CTs), specialty trainees (StRs), dental nurse trainees (DNTs), trainee orthodontic therapists (TOTs)]; 44% (*n* = 19) were primary care dental team members working in the General Dental Service (GDS) or Public Dental Service (PDS)^1^ and of these 63% (*n* = 12) were dentists and the remaining were dental nurses (*n* = 5) and hygienist-therapists (*n* = 2). Sixty-nine percent of the total number of participants (*n* = 24) were female.

**Table 1:** Focus group participants by professional group.

	**Professional group**	**FG1 *n***	**FG2 *n***	**FG3 *n***	**Total *n* (%)**
Trainee focus groups	VDP	3	1	1	5 (31%)
	VDT	–	1	1	2 (13%)
	DCT	2	1	2	5 (31%)
	StR	–	2	–	2 (13%)
	DNT	–	1	–	1 (6%)
	TOT	–	–	1	1 (6%)
	**TOTAL**	5	6	5	16 (100%)
	**Professional group**	**FG1** ***n***	**FG2** ***n***	**FG3** ***n***	**Total** ***n*** **(%)**
Primary care team	Dentist (GDS)	4	4	3	11 (58%)
focus groups	Dentist (PDS)	–	–	1	1 (5%)
	Dental Nurse	3	1	1	5 (26%)
	Hygienist	1	–	–	1 (5%)
	Therapist	–	1	–	1 (5%)
	**TOTAL**	8	6	5	19 (100%)

### Sustainability and Dental Services: Contextual Factors

The COVID-19 pandemic can be understood as a significant macro-level influence not only on our epidemiological reality but also upon the policies and evidence-base within which dentistry operates. In Scotland, in March 2020, all non-urgent dentistry ceased on the directive of the Chief Dental Officer (CDO) as the UK entered a first national lockdown. Dental practices were closed to dental care and instructed to triage patients operating on an Advice: Analgesia: Anti-microbial basis [18]. Patients needing urgent dental treatment were referred to Health Board designated Urgent Dental Care Centres (UDCCs). In a letter dated 20 May 2020 the CDO outlined three key phases of remobilisation: Phase 1: Increasing Capacity of UDCCs, Phase 2: Restarting Dental Practices and Phase 3: Introducing AGPs (Aerosol Generating Procedures) to Dental Practices [19]. No precise timeline was provided at that point however from 22 June 2020 dental practices were able to see NHS patients for face-to-face consultation who were in need of urgent care, using non-aerosol generating procedures and as of 13 July 2020 they were able to see patients for non-aerosol routine care [20, 21].

Dental practitioners and dental care professionals spoke of their concerns surrounding sustainability as a consequence of Government policy. They noted that fallow time between patients, along with the need for social distancing within the practice setting, was reducing patient through-put. Sustainability associated with staff redeployment was also viewed as problematic. In essence, the emergency level response to the pandemic was described by some as resulting in dental services being unsustainable. With little advice being readily available at this time, practitioners voiced how the lack of clear guidance caused considerable uncertainty and how they felt that there should have been greater support from the General Dental Council (GDC): the UK-wide regulator of the dental team. There was concern and unease around litigation and how the GDC would respond to complaints from patients who felt their treatment had been sub-optimal due to the COVID-19 restrictions:

“*[The GDC] …they've given no support to general practice, they've given no support to community either, they just kind of sit on the fence, but if the patient was to complain for any reason I do feel they would be all over the back of you for it and I do think that's unfair because obviously we have to do guidance you know, we have to follow is what we're told”* (Dental Nurse)

At a time where the needs of the wider health care system were emergent and uncertain there were fears voiced around the potential consequences of adapting to these changes, for instance around redeployment:

“*Being told that… you can be redeployed but you have to make sure whatever you're doing when you're redeployed still meets your scope of practice, that you're not going out with your scope of practice or [the GDC] they're going to come for you. They're going to sort you out”* (VDT)

In terms of the remobilisation of dental services, concerns were expressed around how services would manage. While some talked of looking forward to a return to “normal” organization and delivery of services, others highlighted the opportunity, indeed the necessity, for government health policy to change. There was a suggestion that new systems should be devised in order for the unmet backlog of dental need, together with clinical skills and practice resources, to be managed effectively in a pandemic era. What would the ongoing pandemic mean therefore for the future sustainability of dental services?:

“*It's going to be quite difficult to move dentistry back to modern dentistry because there's going to be so much backlog and presumably so much restriction for a considerable period of time”* (GDS Dentist)

“*Is the public dental service going to be sustainable in the long-term?”* (PDS Dentist)

The future of NHS dentistry^2^ was repeatedly called in to question. There were concerns raised about potential changes in dental treatment remuneration and around policy changes which may occur as a result of the pandemic. Would political decision-makers move dentistry toward a “core” group of treatments with everything else paid for privately? Uncertainty regarding the subsequent character of NHS dental care was voiced by many participants along with anxieties associated with dental practice survival within the wider economic and political context. One practice owner felt that there was low political commitment to NHS dentistry and along with an anxiety that changes to remuneration were imminent, described how they were currently grappling with difficult decisions about their future:

“*We're thinking about going private, but… I, I don't want to go private, I, I'd rather remain NHS, but if, our furlough ends when the new SDR [Statement of Dental Remuneration]*^3^ *comes out, which it probably will, that's going to be the time when I think most practices will switch, because we've got to support ourselves”* (GDS Dentist)

Primary care team members voiced significant frustration about not being sufficiently briefed in advance of announcements about changes made by those at the top strategic levels in Scotland, in particular the announcement during October 2020 that as of 1 November 2020 dental contractors would be able to provide “a full range” of dental treatments to NHS patients [22]:

“*I think for me the major frustration has been that we, we've heard about changes that are coming generally after our patients. So the first thing I heard about the fact that things were changing in November was a patient at half past ten saying, “oh, but I'll be able to get my filling done, won't I, in a couple of weeks because it's all changing” and I looked like a complete idiot because I said, “what, what are you, what are you talking about?” And then they said what's been on the news and you know the, the Chief Medical Officer or whoever it was, said dentistry is going back to normal on the 1st of November. And I just had to sort of exchange a glance with my nurse and sort of make some vague noises and say we would get back to them and frantically go and look and see what the news had said”* (GDS Dentist)

Practitioners experienced this situation as both disempowering and highly stressful, and as having the consequence of positioning dentists as “*the bad guys*” in the eyes of the public:

“*[Patients] then think it's us that is somehow gatekeeping the treatment and refusing to do what we're supposed to do”* (GDS Dentist)

“*You've then got the public now getting annoyed with us because we don't know, we can't answer a lot of their questions at the moment”* (Dental Nurse)

This was compounded with a sense that whereas at the start of the pandemic the frequency of information and advice was regular and useful, allowing practitioners to feel “*safe in a way that everybody was doing the same,”* this had diminished over time, creating only more uncertainty and anxiety: “*you're weeks and weeks without hearing any information now, am I still doing what I'm supposed to be doing?”* The disconnect between the profession and the professional leadership, alongside periods of feeling neglected in terms of being provided with regular communications and updates, were seen as inhibiting sustainable practice, in particular around maintaining positive relationships with patients and the wider public.

For those still in training, NES were unable to provide their usual cyclical training programmes, resulting in unprecedented disruption to the trainee workforce. While attempts were made to sustain some training delivery through, for instance, the adoption of Microsoft Teams for selected teaching, overall, the pandemic context acted to severely limit training provision. Trainees spoke of their concerns and anxieties about the sustainability of training in terms of producing required outcomes i.e., in terms of the development of their clinical practice:

“*Are we going to meet training requirements or are we going to be less competent practitioners because training requirements will be reduced because of not being able to see as many patients or as vast a range of patients? You know, does that mean that we're not, we're less competent if we have lower training requirements, or will those not be adjusted? And will we be under more pressure to achieve what others before us had to achieve, but at a different rate?”* (CT3)

There was also a feeling that NES had failed to sufficiently consider or instigate changes to increase the length of training to support those most affected, as put forward by another core trainee:

“*I largely feel that CT training, VT training, there should have been, and could have been, an optional extension of some description and I just think that the powers that be haven't protected, either potentially haven't given that enough, enough of a consideration”* (CT3)

The impact of the pandemic exposed the vulnerability of dental training programmes, and thereby dental workforce planning, to the impact of external crisis events, both in terms of sustainability as the continuation and maintenance of current training provision and the ability of the system to adapt effectively to rapid change. A trainee dental therapist reported feeling “*a bit like we weren't important”* where redeployment opportunities didn't materialize and for those trainees who were redeployed this was described as “*a bit of a lottery”* as to whether this involved clinical work or not. That extensions were provided as an option to StRs in Scotland and to a wider range of trainees in Wales was experienced as hypocrisy. For some there was a sense that for vocational training programmes the “*goal posts”* had been moved and while “*prior to Covid, it was absolutely compulsory to do 12 months of VT”* to attain satisfactory completion, this had been disregarded “*in order to make things fit”* for incoming recruits in the coming September “*instead of focusing on the ones that are leaving and going out into practice and moving on.”*

### Sustainability and Dental Services: Activity Profile

Dental practitioners and dental care professionals spoke of their deep concerns around being unable to sustain their practice to deliver high quality patient care, as well as their fears about the impact that the restrictions on dentistry were having, and would continue to have, on patient and population oral health. “*It feels like it's a bit of a ticking time bomb”* observed one trainee dentist.

There was significant distress about missed and delayed diagnoses for serious disease:

“*By ignoring so many patients which didn't have their, their exams, you may have lost so many cancer patients… I've seen last week a patient and I am very worried about her. There was a lesion there, which I presume it's cancerous, so I will see her again like the guidance in two weeks but like her I may have much more others”* (GDS Dentist)

Concern was also raised about lengthening waiting lists and the growing backlog of patients within the GDS:

“*I'm doing stop gap measures, and so I'm not getting rid of that burden of treatment, I'm just sweeping it all under the carpet and it's getting bigger and bigger and it it's looming there waiting for whenever it is that we can start treating them so we've just got this huge backlog*” (GDS Dentist)

“*Waiting times are going to be absolutely drastic… we're going through the cancelled paeds general anesthetic list trying to sort of prioritise them in some sort of way once the electives are back up and running… and it's a bit of a minefield really”* (CT2)

Anxiety and helplessness about providing what participants considered to be sub-optimal treatments and having to apply “*temporary measures”* on an ongoing basis was also reported. There was a sense that dentistry was “going backwards,” or as one experienced general dental practitioner described it, “*there is an awful lot of blood and vulcanite going on at the moment*.” This was coupled with a feeling that the profession was being forced to act against training and guidelines:

“*We're just sort of putting plasters on everything and I just feel like for some patients you know their dentition is held together with GI [glass ionomer cement] and I just, I have nightmares about it”* (GDS Dentist)

“*During the lockdown we could not do inhalation sedation so I had to refer all of them for GA [general anesthetic], which is not a good experience for the child and like patient safety, the whole point, so all the guidelines and things that we had studied and we were following, it's all turned upside down. It's very, very confusing”* (PDS Dentist)

Contained within a system which could not maintain normal service provision, alongside the ongoing uncertainty about when, or even if, pre-pandemic levels of provision could be re-established, participants were left grappling with how to make sense of this situation for their professional practice. Should they proceed with procedures which go against what they feel is fair or even safe for patients where they are repeatedly advised to do so? In “*extraordinary times”* to what extent should any misgivings be put to the“*back of the head?”* Ethical predicament is also found in one general dental practitioner's consideration of whether strict adherence to “guidance” or “rules” should override other competing moral imperatives, personal or professional: “*starting with the 1st of November you probably will see more than 10 [patients] because, even if we are not supposed to there are so many patients who need to be seen who were neglected over all these months.”*

Some participants indicated their frustration at how the rules would not allow for them to reach vulnerable adults unable to manage their own oral hygiene, including older adults, people with additional support needs, people in the prison system, and children.

“*As the time's going on I'm becoming more and more concerned about my patients, a lot of them are special care whose oral hygiene isn't always maintained particularly well and I just feel I could be seeing them because a lot of them I don't need to use AGP”* (Hygienist)

As the pandemic progressed, the inability of the system to respond sufficiently to the needs of particularly vulnerable groups has acted to severely impede the sustainability of effective services and desirable clinical outcomes for these patients.

“*My team down at the testing centres, they've been going into the care homes and they're doing COVID tests on, on the residents and they can see how filthy their mouths are. I am absolutely desperate to get into the care homes to get some training with the staff”* (Dental Nurse)

“*I really worry, I would like to just go in [to the care homes] with toothbrushes as well and just brush everybody's teeth, if I was allowed, I would”* (GDS Dentist)

Trainee dentists found themselves frustrated by rule inflexibility and being unable to exercise their professional judgement around rule applicability. As one CT3 exclaimed “*I was told I couldn't do it and that was that [but] if I had followed my clinical judgment, it would have been… find a K file [an endodontic file used for mechanical preparation of the root canal system] and do what I need to do, because this gentleman doesn't need an AGP to get him out of pain and keep the teeth*.” There were also examples of how thresholds for treatment produced perverse effects and the consequent challenges this created for maintaining positive relationships with patients:

“*There's kind of a group of patients that don't fit into a category but really need treatment, so patients who have kind of like the start of pulpitis, like lingering sensitivity to cold and things like that, because they've got decay, but because they're not in raging pain the urgent dental centres won't see them for an extirpation because it's such a rigmarole. And obviously that we're just going to have to wait for that to get worse before they're then kind of justifiable for a pulp extirpation, so that's a really difficult conversation to have with patients, that's kind of the most difficult conversation I've had to have with patients really”* (VDP)

For some it also proved challenging to have their patients seen by the UDCCs, as described by a trainee dental therapist:

“*At the beginning we really struggled to try and get patients into the urgent dental care centre, and it took round about 8 weeks from the initial contact from the patient… going through our advice, analgesics or antibiotics and nothing was working…so we've had a lot of patients deregister with the practice because they're unhappy, but it was nothing that we could have done anymore”* (VDT)

A lack of public understanding of the services that were available also acted to impede the delivery of care. One trainee dentist described feeling “*guilt”* about patients not being sufficiently informed: “*I have patients phoning that I'm triaging saying they didn't know they could contact the dentist all this time and that our phones were open this whole time.”* This had led to disastrous consequences for some patients:

“*I mean I just can't believe that people thought there was no help there? We had, I was at theatre one of the times, with a gentleman that tried to take his own tooth out! This is 2020, it's not 1920, come on!”* (Dental Nurse)

Some participants suggested that there may be positive implications for practitioner/patient relationships, insofar as patients may come to appreciate dental services more and the public become more cognisant of the wider skills involved in dental practice. More prevalent however was considerable concern around how years of work in building relationships with patients to ensure continuous dental review, and in particular developing trust with those who are vulnerable or anxious, were at risk of coming undone.

There were also significant concerns around the impact of the pandemic on those who could not afford to access private dental care, and how this may in turn affect future service delivery, for instance, would the PDS have to pick up those patients who could not afford private treatment? Some practitioners described feelings of anxiety and despondency in not being able to offer treatments on the NHS which they could offer privately:

“*I think I'm getting even more anxiety now, because now I feel guilty that I'm saying to patients I can't do this on the NHS but I can do it privately and to me that seems a little bit illogical and patients don't understand this”* (GDS Dentist)

“*The practice that I've been doing my VT in is statistically something like 97% NHS. So none of our patients can get any of the treatment they need, whereas obviously people who are paying privately can get in a lot of practices, can get the treatment that they want at this stage, and it's quite difficult to try and convey that to patients and to explain it because we can't. We can't really explain it…which is really disheartening actually”* (VDP)

The incongruence between NHS and private provision, or as one core trainee described it the “*non-harmonious work going on at the moment*,” was felt to be confusing for practitioners, producing inequalities in terms of the services and level of care available to patients. The notion of an emerging “two-tier” dental system was raised, with implications for the sustainability of treatments on the NHS post-pandemic.

### Sustainability and Dental Services: Organizational Capacity

The whole system disruption to Scottish dental services resulted in significant restrictions in the services offered, the level of care which could be made available and the volume of patients who could be seen. This sudden and dramatic shift in activity profile had immediate and major implications for how work was divided and organized, both across the professional groups and between different service areas. Some established general dental practitioners describe “*sitting at home waiting patiently”* at the start of the pandemic, taking the opportunity to undertake online courses and even finding the situation to be invigorating. Albeit any excitement was short lived:

“*In the first case it was almost thrilling. It was almost exciting. You're in a position where none of the old rules apply. You're making decisions. You're making things happen… That sort of dynamism was really exciting… now, it's dawning on us that that excitement's over and what the hell are we going to do next?”* (GDS Dentist)

For others the pandemic immediately introduced significant challenges, with high levels of anxiety experienced by those working on the frontline emergency response:

“*We had no choice. I was on the front line, I was put, you know, I was told I was going to public health and COVID testing… The team that I went to when I first went there was, there was made of health visitors and school nurses. There were seven of them, six of them, I was sent because six of them were off with COVID. We were all terrified”* (Dental Nurse)

There was a sense that the future burden in terms of additional workload would fall disproportionately on dental nurses and for those nurses who had been deployed to the front line the divergence of experiences had created some resentment:

“*My primary care colleagues were on the same salary sitting at home and I was out there doing 14 hours some days”* (Dental Nurse)

Such divergence of experiences early in the pandemic suggests that dentistry as a health care system was limited in its capability to rapidly mobilize the dental workforce and maximize its potential in a fair and effective way. The medium to longer term capability of the system to undertake workforce planning and retain staff within the sector was also brought in to question. Many participants described how the ongoing uncertainties, and in particular stress about income and employment, were affecting how they were thinking about their professional futures. Some were looking at possibilities for retraining or even considering leaving their profession altogether:

“*I definitely have thought about [whether I still want to be a dentist] multiple times, considering retraining for something else, I know a lot of friends are in the same position as well, or if not, a lot of dentists are now leaning toward trying to get into private dentistry as opposed to NHS, and they see that as the only way forward. So, I think a lot of people, whether they do it or not, are definitely considering leaving the profession, or at least, at least leaving NHS dentistry”* (GDS Dentist)

One early career dentist described actively applying for salaried positions in the PDS in response to the stresses she was experiencing from self-employment, which had been further exacerbated by the pandemic:

“*Throughout COVID you know my stresses come from the fact that my finances were up in the air, so personally for me, I think I want to stay within dentistry, but I would like a salaried position because that is the major source of my stress. So not leaving the profession, but certainly changing what I do because everyone makes such a big deal about how great it is to be a self-employed dentist and I think it's the worst thing in the world. I think it's, it's awful”* (GDS Dentist)

Dental hygienists and therapists reported having no indication of when they would be back at work as “*stressful and disconcerting”* and whilst some were supported through the Coronavirus Job Retention Scheme, for many in the profession the impact on individual income and employment was proving disastrous:

“*Of course I'm a bit lucky because… I was furloughed… but most of my friends, fellow dental therapists and hygienists, I think yeah, the report from [the] British Association of Dental Therapists said that about 40% of the dental therapists and hygienists lost their jobs. So, all that meant that this, this pandemic, has caused a lot of havoc to the families and all those people”* (Therapist)

The financial viability of a career as a hygienist or therapist was also raised by one trainee therapist: “*if you take a percentage cut of the treatment you do to patients with the limited number you'll be able to see you'll really not be able to make enough… to almost stay afloat.”* The sustainability of general practice was also a considerable worry with practice owners voicing their concerns about potentially having to make staff redundant:

“*If we have another six months and another nine months where we can't put anybody into NHS treatment, we will have no income stream in 18 months' time and we will have to lay off staff”* (GDS Dentist)

The difficulties around income and employment had made a considerable impression upon trainees and those early on in their careers. Two early career GDS dentists, who had both started Associate^4^ jobs in March 2020, described how because the Scottish Government funding package was based on 80% of the average income from item of service and patient contributions [23] not having any historical earnings to base payments on had “*sparked a lot of confusion”* with Practitioner Services.^5^ The distress of living with ongoing uncertainty about their expected income and a sense of powerlessness is apparent:

“*It's just very difficult not knowing, you know, you're doing your job and there's nothing you can do about the income side of it. They pay you whatever they pay you and you just have to accept that”* (GDS Dentist)

The threat of unemployment was having a big impact on the anxiety of trainees, in particular those reaching the end of their VT or CT3 year:

“*I won't have a job in four weeks, so I'm a bit worried about the mortgage etc. So I've not been spending any money at all in the last four months”* (CT3)

“*I was terrified if I didn't get a CT job, what I was gonna do… I didn't know anyone in my study group who had a job. Everyone was really struggling to find jobs and I was worried that I was then going to be sort of like late to the party because I've been waiting to find out if I had a CT job. So, I would have literally gone anywhere in the UK. I didn't care, I just wanted to have a salary and feel safe with that”* (VDP)

Trainees described “*frantically searching”* for jobs and being prepared to re-locate to other parts of the UK and/or change specialty. Stories were relayed of friends considering working in supermarkets or deciding to permanently move into different fields, such as health care administration. The usual tacit rules, such as expecting that an Associate role may become available at your VT training practice, no longer applied. The suggestion that dentists should become salaried like medical General Practitioners was met positively, as one VDP said “*I think a lot of people wouldn't be unhappy with that at the moment”* and it was clear that for some, being confronted with precarity of employment as a trainee dentist had come as a shock:

“*I think it's baffling that we're told what 3 or 4 years ago and actually people going into dentistry probably in 2019 were told “Dentistry, it's a remarkably stable career, you will never want for anything in your life. You will earn this and you will do fantastically well, and then you'll have a nice house and you'll have kids and everything will be fine” But actually it's Covid has cast an amazing amount of doubt over that”* (CT3)

The inability to sustain their clinical practice and the associated risk of deskilling was also a major concern for those in training:

“*It's three months that's been taken out of my year-long training, so I feel like there's certain areas in my training that I've got little to no experience in at all”* (VDT)

“*I feel that if anything, the skills that I was becoming competent with in March or at the start of March where I was starting to get a bit of confidence with things, have now been taken from me. I feel like, gosh, I just couldn't do the same things I was doing back in March. I would want a lot more direct supervision and input before I would do that on my return to work”* (CT3)

The medium to longer term impact of the pandemic on the employment experiences and clinical practice of these cohorts of trainees remains to be seen. However, there may be implications for future recruitment if dentistry is no longer viewed as a stable or attractive career choice.

Striking within the data corpus are the regular references to feelings of anxiety and distress induced by the significant uncertainties participants were grappling with due to the impact of COVID-19. The strength of some of the emotions experienced is captured by references to “*it getting to you”* over time, of feeling useless, hopeless, overwhelmed, “*terrified”* and as having “*a big knot in my stomach.”* Participants described the situation as having been a “*nightmare,”* an “*extremely bumpy ride,”* of dreading going into work and going home at night feeling “*just exhausted from it.”*

Some participants expressed anxieties about catching COVID-19 themselves, how to ensure they were protecting their team members and colleagues, and how to avoid transmitting the virus to family members, especially those who may be vulnerable or elderly:

“*Will I get it? Or am I safe? Or am I safe enough? Or am I doing enough to protect me, to protect my family, to protect my team? You don't know”* (GDS Dentist)

Despite these concerns the workplace was often identified as the place where participants felt safest and most in control of their situation. Pre-COVID-19 levels of professional expertise in infection control were viewed as giving dentistry an advantage in ensuring safe practice. However, there were anxieties about the workplace becoming less safe as patient throughput and use of AGPs began to increase.

Some participants reported feeling disconnected and isolated from colleagues, whether due to not being at work at all, or from being kept separate due to safety procedures. Emotional support was sought through informal channels such as by reaching out to friends in the profession for advice and information and by engaging with others on social media. Although online platforms were viewed as having the potential for making positive connections with other professionals they were also felt to directly exacerbate anxiety, as described by one dentist in general practice: “*it's been quite negative, which I guess is a lot of people venting, but it's very easy to get in a spiral of panic.”*

The pandemic acted to heighten the experience of isolation already felt to be prevalent within dentistry. For many, having the opportunity to hear from colleagues about their experiences and to be able to share their own anxieties and feelings were fundamental reasons for volunteering to take part in a focus group. The risk of not having the structures and culture in place to support the mental well-being of dental practitioners and dental care professionals during COVID-19 is captured best in the words of a trainee dental therapist: “*dentistry has one of the highest suicide rates of all professions. There should be such an importance put on mental health within dentistry and there was no support at all.”*

## Discussion

The focus groups took place between July and October 2020 and the findings should be interpreted in that context. This was before the first COVID-19 vaccine was approved in the UK and prior to subsequent lockdown phases in Scotland during autumn 2020 and early 2021.

Olsen's tripartite framework allowed for an in-depth exploration of the factors associated with sustainability of dental service provision and acted as a means for appreciating the external and internal issues associated with sustaining dental services during the current pandemic. A change in one set of the determining factors of sustainability (i.e., context, activity profile, organizational capacity) must be met with change in the others to sustain a service. Certainly, dentistry in Scotland responded to the external crisis with unprecedented action taken across the whole system; non-urgent dentistry ceased and arrangements for the provision of urgent dental care were quickly put in place. In this sense the system did rapidly adapt to a major change in the environmental conditions. However, our findings show that the impact of the pandemic has produced considerable uncertainty regarding the sustainability of dental services in the medium to longer term.

We also found that the pandemic had served to expose the uncertainties practitioners grapple with routinely as they attempt to sustain their NHS dental service delivery. The business of dentistry and the financial viability of running a practice are already established as factors connected with emotional exhaustion among dentists [24]. For those working in the GDS, the pandemic exacerbated the perils of self-employment and running a small business through dramatic loss of income and subsequent complexities of accessing financial support. Tensions around different practice set-ups, in terms of the balance of NHS/private practice commitment were also heightened. Indeed, the need for a suitable long-term post-COVID-19 NHS funding model for dentistry was recognized by the CDO in Scotland with the establishment of a working group in the second half of 2020 [25], however the national consultation initially suggested for Spring 2021 remains pending. A multisite cross-sectional study capturing dentists' perceptions in North America, Europe, Eastern Mediterranean, and Western Pacific regions suggests that the financial effects on practices and the challenge to the sustainability of the business of dentistry have been experienced similarly internationally [26], albeit the offer of stimulus packages and compensation from different governments has varied [26, 27].

The pandemic highlighted the vulnerability of well-established training programmes to a sudden external crisis and various difficulties for sustaining training provision were encountered. Development of comprehensive crisis management plans will be imperative for mitigating future disruption to training, whether from a future pandemic or other external crises. Expansion of the capacity to deliver digital learning and increased use of simulation training for clinical and non-clinical competencies will need to be prioritized, as shall the development of competence frameworks which better support trainees to engage productively in roles outside of their dental capabilities, for instance when redeployed [28]. Experiences of employment insecurity, under-employment and in some cases unemployment or redundancy are likely to stay with those affected for the rest of their careers [29]. For trainees, being unable to secure employment challenged core beliefs about the relative security of their career choice. For others, such as associate dentists and dental hygienists/therapists, the pandemic brought to a head already existing concerns around employment status and financial viability. While precarious working is usually considered in relation to elementary, caring and leisure occupations, there is the potential for precariousness in any employment based on a range of risk factors and aspects of the employment context [30]. Going forward a push for more salaried roles should perhaps be anticipated [29].

The disconnect experienced between the profession and the professional leadership, as well as a deepening distrust of the regulatory body, could have potentially long-term and damaging consequences if not carefully addressed. Trust is associated with the long-term willingness of staff to remain with an organization and expend effort in achieving its goals [31]. It is also critical in the successful implementation of change programmes [32]. Retaining the dental workforce and building support around a new and sustainable post-pandemic vision will require trust to be re-built with those who have lost confidence in the dental leadership. In light of Scottish Government plans to abolish all NHS dentistry charges over the course of this parliament [33], and further promises around reforming funding arrangements, this is a pressing matter.

Greenberg et al. contend that “*whether someone develops a psychological injury or experiences psychological growth is likely to be influenced by the way that they are supported before, during, and after a challenging incident*” [34]. Our findings bring in to sharp focus the diversity of challenges, confusions and uncertainties experienced by dental practitioners and dental care professionals during the COVID-19 pandemic. The focus group methodology allowed participants to share and reflect upon their feelings of anxiety, fear, anger, and even shame, and while the level of distress varied from individual to individual, the need for suitable and ongoing measures to be put in place to support mental well-being during COVID-19 and beyond is apparent. Intervention at the level of the individual is necessary, however may not be sufficient. Dentists working in the UK exhibited burnout and low well-being before the pandemic arrived and high levels of stress in the dental profession are well-documented [35]. Toon et al. found that work content stress, productivity stress, patient-led stress and regulatory stress all drive burnout within general dental practitioners. They highlight how the latter three of these drivers are largely environmental conditions that shape the context within which dentists work, thereby implying that key areas of stress that cause burnout are in fact outside of the full control of the dentist. We concur with these authors, and others such as Gallagher et al., that solutions to high levels of stress and burnout must therefore extend beyond support for the individual. Urgent action is needed at policy level with implementation of system-wide interventions, which emphasize cultural change within the sector, for the long-term sustainability of high-quality dental services in Scotland [36, 37].

One concept which has garnered particular interest in relation to healthcare workers during the pandemic is “moral injury.” This can be understood as “*the psychological distress that results from actions, or the lack of them, which violate someone's moral or ethical code”* [34]. This distress is understood to potentially contribute to the development of mental health difficulties, such as depression, post-traumatic stress disorder, and suicidal ideation [34]. The concept provides a potentially useful lens for trying to understand how dental practitioners and dental care professionals have experienced and continue to experience carrying out their work in situations which are at times extraordinarily demanding and even traumatic [38]. We have identified in this study evidence of how actions taken (e.g., multiple temporary fixes) and the inability to take action (e.g., being unable to access care home residents), produced in participants feelings of helplessness and uselessness. The empirical literature on moral injury regarding dentistry is currently very limited and there is scope for further work in this area.

Limitations of this study must be acknowledged. We recognize that those who chose to volunteer to participate in a focus group which involved discussing their anxiety and concerns regarding their current job role and future career may not be representative of the baseline survey sample, or indeed the wider population. We feel however that the focus group method allowed for the generation of rich and insightful data which could not have been accessed by survey methods alone and adds both depth and comprehensiveness to the quantitative data collected as part of the CAREER research programme. We held mixed rather than single professional groups as we were keen to identify any divergences in opinion and allow for dialogue about differing experiences between participants. We are mindful however of how power dynamics at play in the workplace may replicate in the research environment and despite the best efforts of the facilitators it is possible that some individuals would have felt more comfortable in a single professional group.

## Conclusion

This study explored qualitatively how the COVID-19 pandemic affected dental practitioners and dental care professionals in training and in primary care teams in terms of their lived experiences of uncertainty and attempts to sustain dental services during COVID-19. The capability of the dental health care system to deliver high quality care as well as meet a growing backlog of dental need and manage this effectively in a pandemic era is called in to serious question and doubts are cast around the longer-term sustainability of NHS dentistry. The pandemic also served to expose uncertainties practitioners grapple with routinely as they attempt to sustain their NHS dental service delivery, for instance regarding the financial viability of running a practice as a business and ongoing difficulties with the funding model. The study found a disconnect between the profession and the professional leadership, as well as a deepening of distrust in the regulatory body. It is critical that trust is rebuilt to enable the retention of the dental workforce and the building of a new and sustainable post-pandemic vision for dentistry in Scotland. This vision needs to include suitable and ongoing measures for supporting the mental well-being of dental practitioners and dental care professionals, at both the level of the individual and the wider team, and in terms of policy and system-wide interventions.

## Data Availability Statement

The datasets presented in this article are not readily available because the Ethical review board instructed that the data collected remain within the NHS Education for Scotland repository and jurisdiction. Requests to access the datasets should be directed to sdpbrn@nes.scot.nhs.uk.

## Ethics Statement

The studies involving human participants were reviewed and approved (18th May 2020) by the University of Dundee Nursing and Health Sciences and Dentistry Research and Ethics Committee (Reference: UOD/SDEN/STAFF/2020/013- Freeman). The participants provided their written informed consent to participate in this study.

## Author Contributions

RF, GH, and JC conceived the study. JC secured support for the study. RF and JK led the focus group facilitation with support from LB, MA, and SY. JK led the data analysis and the drafting of the manuscript. RF, LB, MA, and SY contributed to the data analysis. All authors contributed to the study design and contributed to drafts and approved the final manuscript for submission.

## Funding

NHS Education for Scotland provided the open access publication fee.

## Conflict of Interest

The authors declare that the research was conducted in the absence of any commercial or financial relationships that could be construed as a potential conflict of interest.

## Publisher's Note

All claims expressed in this article are solely those of the authors and do not necessarily represent those of their affiliated organizations, or those of the publisher, the editors and the reviewers. Any product that may be evaluated in this article, or claim that may be made by its manufacturer, is not guaranteed or endorsed by the publisher.

## Footnotes

^1^The NHS General Dental Service is provided by independent, self-employed dentists who contract with their local Health Board to provide NHS primary care dental services to patients. The Public Dental Service employs salaried dentists to provide NHS primary care dental services to patients who cannot access a GDS dentist.

^2^In the UK dentists may undertake work under the NHS or privately. Unlike the majority of NHS services most adult patients have to pay a contribution towards the cost of their NHS dental care, however NHS provision is often significantly cheaper than the cost of private dentistry.

^3^The Statement of Dental Remuneration contains a scale of fees describing remuneration for care and treatment provided to NHS patients and details of allowances in Scotland.

^4^The majority of dentists in general practice work as self-employed associates. Associates make regular payments to the practice owner/principal dentist in return for the use of the practice premises, equipment, materials and staff.

^5^Practitioner Services is the national body in Scotland that authorises payments to primary care dentists in terms of the SDR.
