# Supplementary material for: Uncertainty and Fears Around Sustainability: A Qualitative Exploration of the Emotional Reactions of Dental Practitioners and Dental Care Professionals During COVID-19
Source: Front Oral Health. 2022 Jan 21;2:799158. doi: 10.3389/froh.2021.799158 (PMC8813960; doi:10.3389/froh.2021.799158)
Supplement: Supplementary file 1 [file Data_Sheet_1.PDF]

CAREER (PCT)

CAREER (PCT): COVID-19: Dental health professionals in the Primary Care Team: a longitudinal study of uncertainties, **A**nxieties and p**RE**paredn**E**ss for p**R**actice

The purpose of this research is to explore how the COVID-19 pandemic is affecting the dental professionals preparedness for practice, their feelings of uncertainty and anxieties, given the new roles and responsibilities they may find themselves facing during the pandemic and also as routine dental services resume. This project will also investigate how the pandemic, and the cessation of routine dental treatment, is affecting dental education and training. The findings will inform the future provision of educational support and training for dental professionals, including primary care dental teams.

In order for you to participate in this survey, please complete the consent form below.

Informed Consent Form

Section 1. Taking part in the study

1) * 1. I have read the Participant Information Sheet, or it has been read to me. I have been able to ask questions about the study and my questions have been answered to my satisfaction.

○ Yes ○ No

2) * 2. I consent voluntarily to be a participant in this study and understand that I can refuse to answer any question and I can withdraw from the study at any time, without having to give a reason. I also understand that if I withdraw from the study, any data already collected for the study will be kept anonymously and used for the study unless I ask for it to be removed. This will include research publications.

○ Yes ○ No

3) * 3. I understand that taking part in the study involves answering questionnaires related to my levels of anxiety, uncertainty and preparedness for practice during the COVID-19 pandemic.

○ Yes ○ No

4) * 4. I would like to volunteer to keep a weekly diary for the duration of the study related to my emotional state and routine.

○ Yes ○ No

5) * 5. I would like to volunteer to participate in a focus group which will involve discussing my anxiety and concerns regarding my current job role during the COVID-19 pandemic and my future career.

○ Yes ○ No

6) * 6. I understand that this focus group will be video-recorded.

○ Yes ○ No

Section 2. Use of the information in the study

7) * 7. I understand that all personal data collected will be managed in line with General Data Protection Regulations (GDPR) 2018. For more information, please see the NES Privacy Policy document http://www.nes.scot.nhs.uk/privacy-and-data-protection.aspx

○ Yes

○ No

8) * 8. I understand that information I provide will be used in research outputs, including articles in peer-reviewed journals and a report to NHS Education for Scotland and the Chief Dental Officer for Scotland.

○ Yes

○ No

9) * 9. I understand that personal information collected about me will not be shared beyond the study team.

○ Yes

○ No

10) * 10. I agree that anonymised direct quotes can be used in research outputs.

- Yes
- No

Section 3. Future use and reuse of the information by others

11) * 11. I agree that confidential information about myself collected for this study may be used in research in the future. Any information which identifies me will be removed before it is shared.

- Yes
- No

Section 4. Signature

12) * 12. Name: Please enter in space provided

13) * 13. GDC number: Please enter in space provided

14) * 14. Date of consent: Please enter in space provided

15) * 15. Please provide your email address in the space provided. This will be used to contact you with the follow-up questionnaire in 6 months and if you have volunteered to keep a weekly diary and/or join a focus group.

Section 5. Study contact details for further information

Project Lead: Professor Ruth Freeman, School of Dentistry, University of Dundee

Project Specialist Research Lead: Jennie Knights, Dental Clinical Effectiveness, NHS Education for Scotland

16) 16. Email: sdpbrn@nes.scot.nhs.uk

17) * 17. Please insert your GDC number in the space provided.

Most of the questions require you to select an answer, but there are also a number of text boxes that we hope you will use. **Please do not take too long over your replies, your immediate reaction to each question is likely to be more accurate than a long thought out response.**

18) 18. Q1) In the current COVID-19 pandemic, how well are you prepared for:

	Unprepared	Not well prepared	Prepared	Well prepared	Extremely well prepared
Managing your health including stress	○	○	○	○	○
Coping with uncertainty in general	○	○	○	○	○
Coping with uncertainty about future and career job prospects	○	○	○	○	○
Coping with financial insecurities	○	○	○	○	○
Understanding the purpose and practice of appraisal	○	○	○	○	○
Engaging in self critique of practice and clinical encounters	○	○	○	○	○
Engaging in self-directive life-long learning	○	○	○	○	○
Coping with GDC governance and training requirements	○	○	○	○	○
Maintaining good quality of care	○	○	○	○	○
Taking part in clinical governance	○	○	○	○	○
Reducing the risk of cross-	○	○	○	○	○

	Unprepared	Not well prepared	Prepared	Well prepared	Extremely well prepared
infection					
Organisational decision making	○	○	○	○	○
Ensuring patient safety	○	○	○	○	○
Reporting and dealing with error and safety incidents	○	○	○	○	○

19) 19. Q2) Over the last 2 weeks, how often have you been bothered by any of the following problems?

	Not at all	Several days	More than half the days	Nearly every day
Little interest or pleasure in doing things	○	○	○	○
Feeling down, depressed or hopeless	○	○	○	○

20) 20. Q3) Please answer each of the items below in relation to the COVID-19 pandemic:

	Never	A few times a year	Once a month	A few times a month	Once a week	A few times a week	Every day
I feel emotionally drained from my work	○	○	○	○	○	○	○
I feel used up at the end of the work day	○	○	○	○	○	○	○
I feel fatigued when I get up in the morning and have to face another day on the job	○	○	○	○	○	○	○
Working with people all day is really a strain for me	○	○	○	○	○	○	○
I feel burned out from my work	○	○	○	○	○	○	○
I feel frustrated by my job	○	○	○	○	○	○	○
I feel I'm working too hard on my job	○	○	○	○	○	○	○
Working with people directly puts too much stress on me	○	○	○	○	○	○	○
I feel like I am on the end of my rope	○	○	○	○	○	○	○

21) 21. Q4) Please answer each of the items below in relation to the COVID-19 pandemic:

	Never	A few times a year	Once a month	A few times a month	Once a week	A few times a week	Every day

	Never	A few times a year	Once a month	A few times a month	Once a week	A few times a week	Every day
I feel I treat some patients as if they were impersonal 'objects'	○	○	○	○	○	○	○
I've become more callous towards people since I took this job	○	○	○	○	○	○	○
I worry that this job is hardening me emotionally	○	○	○	○	○	○	○
I don't really care what happens to some patients	○	○	○	○	○	○	○
I feel patients blame me for some of their problems	○	○	○	○	○	○	○

22) 22. Q5) Please read each item and then indicate how distressing each issue has been for you DURING THE PAST SEVEN DAYS with reference to the COVID-19 pandemic and effects on your work/practice.

	Not at all	A little bit	Moderately	Quite a bit	Extremely
Any reminder brought back feelings about it	○	○	○	○	○
I had trouble staying asleep	○	○	○	○	○
Other things kept making me thinking about it	○	○	○	○	○
I felt irritable and angry	○	○	○	○	○
I avoided letting myself get upset when I thought about it or was reminded of it	○	○	○	○	○
I thought about it when I didn't mean to	○	○	○	○	○
I felt as if it hadn't happened or wasn't real	○	○	○	○	○
I stayed away from reminders of it	○	○	○	○	○
Pictures about it popped into my mind	○	○	○	○	○
I was jumpy and easily startled	○	○	○	○	○
I tried not to think about it	○	○	○	○	○
I was aware that I still had a lot of feelings about it but I didn't deal with them	○	○	○	○	○
My feelings about it were kind of numb	○	○	○	○	○
I found myself acting or feeling like I was back at that time	○	○	○	○	○
I had trouble falling asleep	○	○	○	○	○
I had waves of strong feelings about it	○	○	○	○	○
I tried to remove it from my memory	○	○	○	○	○

	Not at all	A little bit	Moderately	Quite a bit	Extremely
I had trouble concentrating					
Reminders of it caused me to have physical reactions such as sweating, trouble breathing, nausea or a pounding heart					
I had dreams about it					
I felt watchful and on-guard					
I tried not to talk about it					

DEMOGRAPHIC QUESTIONS

23) 23. Q1) Age in years (Please advise if you would prefer not to say)

24) 24. Q2) Sex

- Male
- Female
- Prefer not to say

25) 25. Q3) Professional Group:

- Dentist (GDS)
- Dentist (PDS)
- Dental Nurse
- Hygienist
- Therapist
- Hygienist/Therapist
- Orthodontic Therapist

26) 26. Q4) In which type of practice do you work?:

- Fully NHS
- Mostly NHS
- Equal NHS/Private
- Mostly Private
- Fully Private

27) 27. Q5) During the COVID-19 pandemic, have you been re-deployed.

- Yes
- No

28) 28. Q5a) If you answered yes to Q5, please advise where you were redeployed to in the space provided:

29) 29. Q6) Where do you want to be professionally in 5-years-time? Please state:

30) 30. If you would like to add any information regarding this questionnaire or your experiences during the COVID-19 pandemic, please comment in the space provided:

[Send]

100 % completed

© Copyright www.questback.com. All Rights Reserved.
Trial Essentials for free - Click here to create your survey today.
